# Risk assessment of malaria transmission at the border area of China and Myanmar

**DOI:** 10.1186/s40249-017-0322-2

**Published:** 2017-07-06

**Authors:** Benyun Shi, Jinxin Zheng, Hongjun Qiu, Guo-Jing Yang, Shang Xia, Xiao-Nong Zhou

**Affiliations:** 1 School of Cyberspace, Hangzhou Dianzi University, Hangzhou, 310018 China; 2 Jiangsu Institute of Parasitic Diseases, Wuxi, 214064 China; 3 National Institute of Parasitic Diseases, Chinese Center for Disease Control and Prevention, Shanghai, China; 4 Key Laboratory of Parasite and Vector Biology, MOH; WHO Collaborating Center for Tropical Diseases, Shanghai, 200025 China

## Abstract

**Background:**

In order to achieve the goal of malaria elimination, the Chinese government launched the National Malaria Elimination Programme in 2010. However, as a result of increasing cross-border population movements, the risk of imported malaria cases still exists at the border areas of China, resulting in a potential threat of local transmission. The focus of this paper is to assess the *Plasmodium vivax* incidences in Tengchong, Yunnan Province, at the border areas of China and Myanmar.

**Methods:**

Time series of *P. vivax* incidences in Tengchong from 2006 to 2010 are collected from the web-based China Information System for Disease Control and Prevention, which are further separated into time series of imported and local cases. First, the seasonal and trend decomposition are performed on time series of imported cases using Loess method. Then, the impact of climatic factors on the local transmission of *P. vivax* is assessed using both linear regression models (LRM) and generalized additive models (GAM). Specifically, the notion of vectorial capacity (VCAP) is used to estimate the transmission potential of *P. vivax* at different locations, which is calculated based on temperature and rainfall collected from China Meteorological Administration.

**Results:**

Comparing with Ruili County, the seasonal pattern of imported cases in Tengchong is different: Tengchong has only one peak, while Ruili has two peaks during each year. This may be due to the different cross-border behaviors of peoples in two locations. The vectorial capacity together with the imported cases and the average humidity, can well explain the local incidences of *P. vivax* through both LRM and GAM methods. Moreover, the maximum daily temperature is verified to be more suitable to calculate VCAP than the minimal and average temperature in Tengchong County.

**Conclusion:**

To achieve malaria elimination in China, the assessment results in this paper will provide further guidance in active surveillance and control of malaria at the border areas of China and Myanmar.

**Electronic supplementary material:**

The online version of this article (doi:10.1186/s40249-017-0322-2) contains supplementary material, which is available to authorized users.

## Multilingual abstracts

Please see Additional file 1 for translation of the abstract into the five official working languages of the United Nations.

## Background

Malaria is one of the important vector-borne infectious diseases that seriously endanger the public health and affect the socio-economic development [1, 2]. For example, malaria caused approximately 216 million infections and about 438 000 deaths worldwide in 2015 [3]. Many initiatives and programmes have been launched to help develop implementable action plans for malaria control and elimination, such as the Global Malaria Initiatives, the U.S. President’s Malaria Initiative and the Lubombo Spatial Development Initiative [4]. Previous experiences emphasize that border areas should be a focus for malaria control activities given the high intensity of both formal and informal movement of people and goods across borders [5]. For exmaple, in the Greater Mekong Subregion, malaria transmission is largely confined to the border areas between Burma, Cambodia, and Thailand [6]. On the one hand, the cross-border movement of populations can introduce malaria cases from high-transmission areas into previously low-transmission or malaria-free areas [7–10]. On the other hand, the imported cases may cause the recurrence of malaria when the environment is suitable for local transmission. Therefore, to achieve global malaria eradication, one of the most important research agenda is to strengthen regional intercountry collaborations, especially the cross-border collaborations [11].

Historically, malaria was extensively endemic in China, especially in the 1960s and 1970s [12]. Since the Chinese government launched the National Malaria Elimination Programme (NMEP) in 2010, great progress have been made to achieve the elimination of malaria. During the implementation of the NMEP from 2010 to 2014, local malaria incidence has declined continuously, except for the border areas of Yunnan Province, China [13]. Yunnan Province shares a 4 061-kilometer borderline with Myanmar, Laos, and Vietnam. Because the border areas are mostly mountainous, crossing the border becomes very easy. It has been estimated that there are several millions of people belonging to the mobile and migrant population (MMP), who may cross the border every year [14]. In 2011, the number of malaria cases in Yunnan Province was the highest in China and accounted for 34% of the total cases in the country [15]. However, according to the malaria report in 2013, about 91.2% malaria cases in Yunnan Province are imported from neighboring countries, among which 79.6% were caused by the *Plasmodium vivax* parasite [16]. Because the imported cases can result in local transmission of malaria in a suitable environment, it becomes one of the biggest obstacles for nationwide elimination of the disease in China [17, 18].

In this paper, we focus on assessing the transmission risk of *P. vivax* in Tengchong County, Yunnan Province, China, which is at the border area of China and Myanmar. Existing studies have shown that the impedance of malaria control in Tengchong are more likely due to the increase of formal and informal human movement across China-Myanmar border regions [19, 20]. Different cross-border activities may result in different temporal patterns of imported cases (e.g., the number of peak transmission seasons). For example, people engaged in frontier trade may frequently cross the border; while local farmers may go to Myanmar for logging or mining during the slack seasons in farming. In this case, to take a step forward to malaria elimination and prevent the recurrence of malaria in China, it would be better to analyze the temporal patterns of imported cases so as to further investigate the purpose of human cross-border activities. By doing so, active surveillance and target interventions can be planned and implemented.

From the perspective of disease epidemiology, the natural transmission of *P. vivax* depends on the interactions between female anopheles mosquitoes and human beings. Existing studies have shown that the ability of mosquitoes to transmit *P. vivax* can be affected by a series of biological factors, such as the daily survival rate of mosquitoes and the sporogonic cycle of sporozoits in their bodies [21]. Researchers have revealed that meteorological factors can also significantly affect mosquito population as well as their biological cycles [22, 23]. Specifically, a vectorial capacity model has been proposed to estimate the malaria transmission potential, which takes into consideration the impact of temperature and rainfall on the bionomics of mosquitoes and the extrinsic incubation period of parasites [24]. Taking into consideration both human cross-border movements and local environmental factors, the objective of this work is twofold: (i) to investigate the temporal patterns of imported cases so as to guide the implementation of active surveillance; and (ii) to evaluate the impact of meteorological factors on the local transmission risk of *P. vivax* in Tengchong County.

The analysis procedures of this paper is organized as follows. First, we demonstrate the different temporal patterns of imported *P. vivax* cases in Tengchong and Ruili counties by performing seasonal and trend decomposition using Loess method. Then, we assess the relationship between local *P. vivax* cases and meteorological factors using both linear regression models (LRM) and generalized additive models (GAM). Moreover, to characterize the nonlinear relationship between *P. vivax* transmission potential and meteorological factors, the notion of vectorial capacity (VCAP) is adopted, which is defined as “the number of potentially infective contacts an individual person makes, through vector population, per unit time [25].” Both the LRM and GAM results show that the VCAP together with the imported cases can better explained the local infections of *P. vivax*. Specifically, we verify that the maximum daily temperature is more suitable to calculate VCAP than the minimal and average temperature in Tengchong County.

## Methods

### Data collection

In China, there is a sound surveillance system for infectious diseases, namely the China Information System for Disease Control and Prevention (CISDCP), where malaria cases are reported daily from each public health facility [26]. According to the Action Plan of China Malaria Elimination, it is obligatory for any medical institutions and hospitals to report clinically confirmed infection cases into the system. In this case, even though the underreporting of *P. vivax* infections is still unavoidable, the number of missing report should be negligible. Time series of *P. vivax* cases in Tengchong and Ruili are collected and aggregated from CISDCP on a daily basis ranging from 2006 to 2010, where imported cases (*I*_*P*.*v*_) are discriminated from local infections (*L*_*P*.*v*_) by doctors or public health investigators through face-to-face case studies. Accordingly, meteorological data are collected from China Meteorological Administration on a daily basis, which include the minimum temperature (*T*_*min*_), average temperature (*T*_*avg*_), maximum temperature (*T*_*max*_), rainfall (*R*), average humidity (*H*_*avg*_), and minimum humidity (*H*_*min*_).

### Vectorial capacity

The notion of vectorial capacity (VCAP) has been extensively used to assess malaria transmission potential based on meteorological factors [25, 27, 28]. The VCAP is derived from the basic reproductive number calculated based on the Macdonald model [29]. Mathematically, it is formulated as follows:

1 $$ V = \frac{-\left(m a^{2}\right)p^{n}}{\ln(p)},  $$

where *m* represents the equilibrium mosquito density per person, *a* is the expected number of bites on human beings per mosquito per day, *p* is the probability of a mosquito surviving through one whole day, and *n* is the entomological incubation period of malaria parasites. Based on the study of Ceccato et al. in [24], all these parameters are dynamically dependent on the temperature *T* and rainfall *R*. Specifically, we have *m*=100∗*R*, *a*=0.7/*g*, *g*=[36.5/(*T*+2.0−9.9)]+0.5, *p*=0.5^1/*g*^, and *n*=105/2∗[36.5/(*T*+2.0−9.9)]/*g*+*T*−18. For more details about the model parameters, please refer to the Table 2 in [27]. With respect to different temperature (resp., *T*_*min*_, *T*_*avg*_, and *T*_*max*_), we have different values of VCAP (resp., *V*_*min*_, *V*_*avg*_, and *V*_*max*_), where the subscripts correspond to temperature *T*_*min*_, *T*_*avg*_, and *T*_*max*_. In this paper, we exam the suitability of *T*_*min*_, *T*_*avg*_, and *T*_*max*_ to estimate the transmission potential of *P. vivax* in Tengchong County.

### Analysis methods

To reveal the temporal patterns of imported cases, the seasonal and trend decomposition are performed on the daily time series using Loess method. To a certain extent, the *seasonality* reveals the pattern of human cross-border movements, which can guide the active surveillance and control of imported cases from neighboring countries. While the *trend* reflects the strength of disease intervention implemented by public health authorities, which are further adopted in our methods to model the risk of local malaria transmission.

To explore the relationship between local transmission risk of *P. vivax* and associated environmental factors, time series of local *P. vivax* incidences in Tengchong are fitted by meteorological factors using both linear regression models (LRM) and generalized additive models (GAM). Since the number of daily *P. vivax* incidences are very small, in this study, both imported and local cases are aggregated biweekly. Accordingly, the time series of environmental factors are also averaged over 14 days. This is reasonable because although the *P. vivax* parasites may stay dormant for a long time, the incubation period of *P. vivax* is usually from 12 to 20 days. The Pearson coefficient is first calculated to measure the linear correlation between each pair of these meteorological factors. Then, several LRM and GAM models are assessed to predict the expected number of local incidences (*L*_*P*.*v*_) from the meteorological factors and the number of imported cases (*I*_*P*.*v*_). Mathematically, the following models are proposed:

$$\begin{array}{*{20}l} \text{LRM-1:} & L_{P.v} = \beta_{0} + \beta_{1} T_{min} + \beta_{2} T_{avg} + \beta_{3} T_{max}\\ &\qquad\quad+ \beta_{4} R + \beta_{5} H_{avg} + \beta_{6} H_{min} + \beta_{7} I_{P.v},\\ \text{LRM-2:} & L_{P.v} = \beta_{0} + \beta_{1} T_{avg} + \beta_{2} R + \beta_{3} H_{avg} + \beta_{4} I_{P.v},\\ \text{GAM-1:} & log(L_{P.v}) = \beta_{0} + f_{1}\left(T_{min}\right) + f_{2}(T_{avg})\\ &\qquad\qquad\quad+ f_{3}(T_{max}) + f_{4}(R) + f_{5}(H_{avg})\\ &\qquad\qquad\quad+ f_{6}(H_{min}) + f_{7}(I_{P.v}),\\ \text{GAM-2:} & log(L_{P.v}) = \beta_{0} + f_{1}(T_{avg}) + f_{2}(R) + f_{3}(H_{avg})\\ &\qquad\qquad\quad+ f_{4}(I_{P.v}), \end{array} $$

The reason to propose LRM-2 and GAM-2 is that there exists significant correlation among *T*_*min*_, *T*_*avg*_, and *T*_*max*_, and between *H*_*min*_ and *H*_*avg*_.

In addition, another three assessment models are built upon the notion of vectorial capacity *V*, which integrates both temperature and rainfall. Taking into consideration the strength of malaria control implemented by public health authorities, a linear trend of time series of *P. vivax* incidences is combined with VCAP to reflect the risk of *P. vivax* infection. To assess the suitability of *V*_*min*_, *V*_*avg*_, and *V*_*max*_ for estimating the *P. vivax* transmission potential, the following GAM models are proposed:

$$\begin{array}{*{20}l} \text{GAM-V-MIN:} & log(L_{P.v}) = \beta_{0} + f_{1}((a+bt)V_{min})\\ &\qquad\qquad\quad+ f_{2}(H_{avg}) + f_{3}(I_{P.v}),\\ \text{GAM-V-AVG:} & log(L_{P.v}) = \beta_{0} + f_{1}((a+bt)V_{avg})\\ &\qquad\qquad\quad+ f_{2}(H_{avg}) + f_{3}(I_{P.v}),\\ \text{GAM-V-MAX:} & log(L_{P.v}) = \beta_{0} + f_{1}((a+bt)V_{max})\\ &\qquad\qquad\quad+ f_{2}(H_{avg}) + f_{3}(I_{P.v}). \end{array} $$

where (*a*+*bt*) is the trend obtained from the seasonal and trend decomposition of *P. vivax* incidences. All these models will be assessed based on the available dataset in Tengchong County.

## Results

### Time series decomposition of *P. vivax* cases

**Fig. 1:**
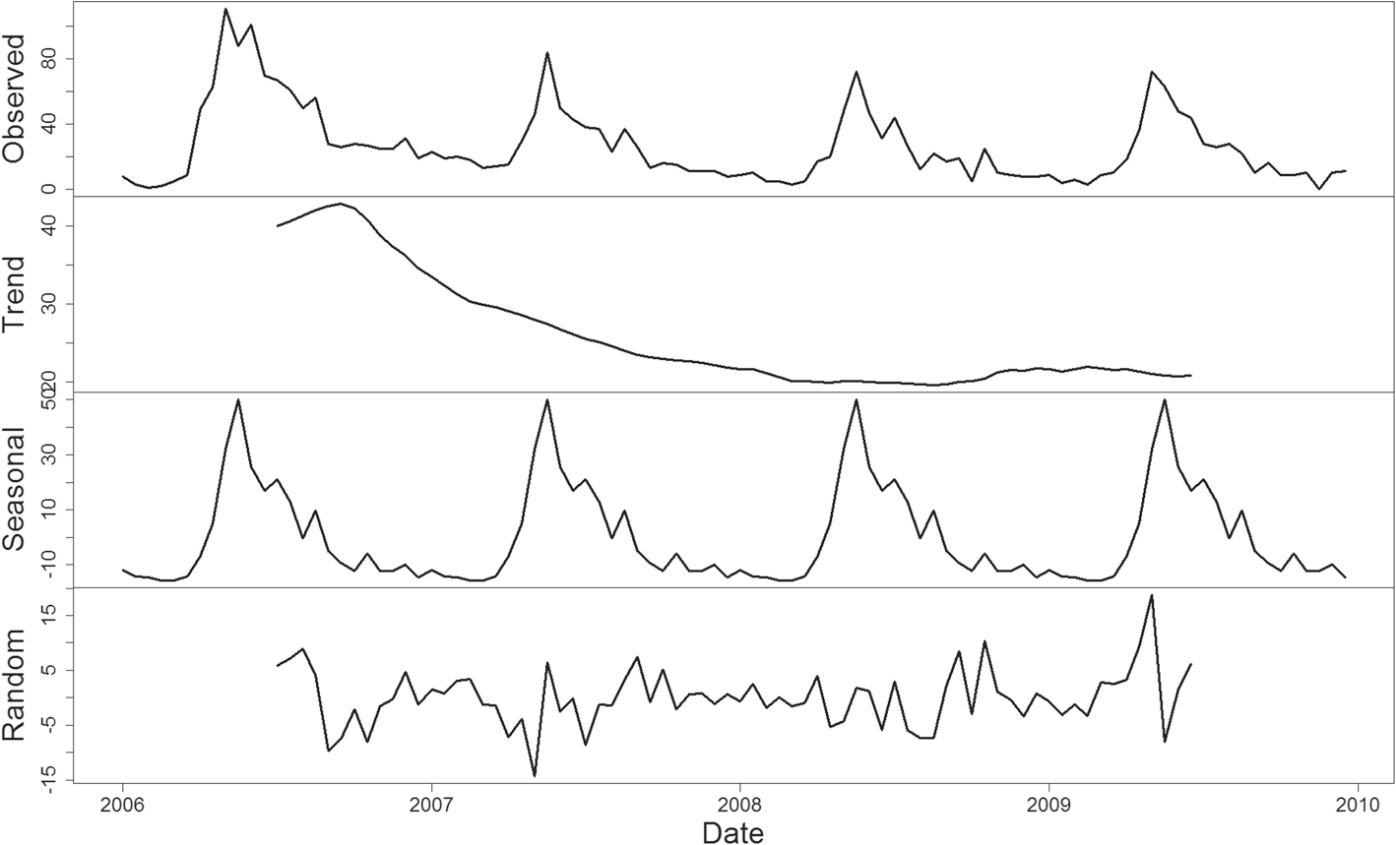
The seasonal and trend decomposition of imported vivax malaria cases in Tengchong Countiy. The seasonality reveals the pattern of human cross-border movements, while the trend reflects the strength of malaria intervention and control. It can be observed that there is only one peak of imported cases each year

**Fig. 2:**
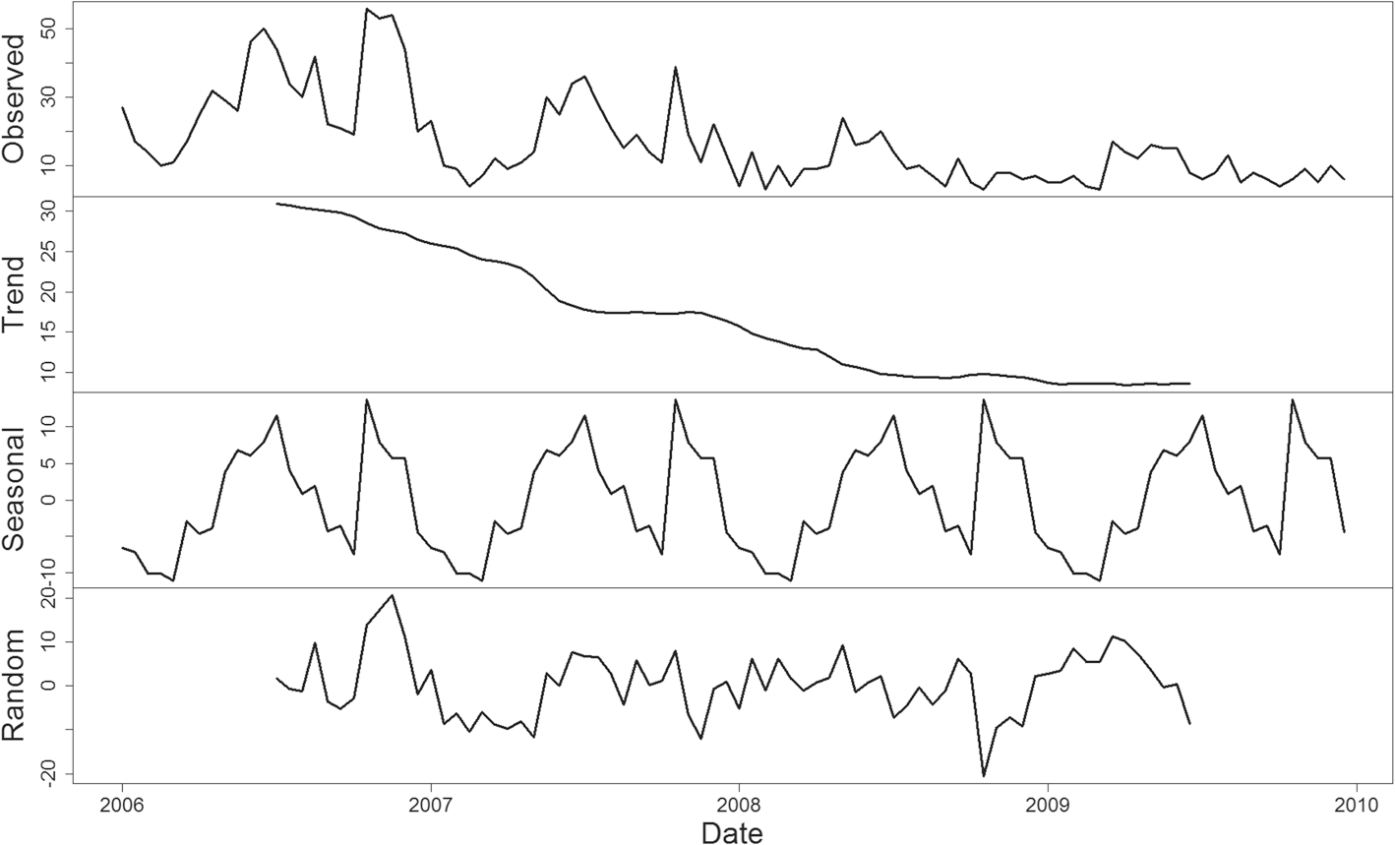
The seasonal and trend decomposition of imported vivax malaria cases in Ruili County. The seasonality reveals the pattern of human cross-border movements, while the trend reflects the strength of malaria intervention and control. It can be observed that there are two peaks of imported cases each year

The seasonal and trend decomposition are performed on time series of imported *P. vivax* cases using Loess method. To a certain extent, the seasonality reveals the pattern of human cross-border movements, while the trend reflects the strength of malaria intervention and control. A comparison is conducted between Tengchong and Ruili, where Ruili is another malaria-endemic county at the border area of China and Myanmar. Figure 1 shows the decomposition results in Tengchong County, and Fig. 2 shows the decomposition results in Ruili County. It can be observed that the trend of imported *P. vivax* cases in both counties declines almost linearly, reflecting the effectiveness of the implemented malaria intervention policy. However, the patterns of seasonality is totally different in the two counties: Tengchong has only one peak every year, while Ruili has two peaks. This may be due to different human cross-border activities in these two counties. Comparing to Ruili, Tengchong is a little far away from the customs on the China-Myanmar border. Detailed investigations in Tengchong show that most farmers in the same villages often go to Myanmar in groups during the slack season, and come back before the busy seasons. Such activities happens once a year. However, due to the lack of manpower and resources, such investigations in Ruili are not put into effect. Even so, the observations provide an insight into the implementation of active surveillance on imported cases to further investigate the motivations of their cross-border activities other counties at the border area. The reason is that different cross-border activities may result in different seasonal patterns of imported cases, which further trigger different risks of infection at different time of a year. Therefore, targeted intervention strategies are required for counties with different patterns of imported cases.

### The relationship between local infections and meteorological factors

To reveal the relationship between local infections and meteorological factors, the Pearson correlation coefficients between each pair of meteorological factors are first calculated, where daily time series of *T*_*min*_, *T*_*avg*_, *T*_*max*_, *R*, *H*_*avg*_ and *H*_*min*_ with lag zero are used. Table 1 shows the correlation matrix between meteorological factors in Tengchong County. Strong linear correlation can be found among *T*_*min*_, *T*_*avg*_, and *T*_*max*_, as well as between *H*_*avg*_ and *H*_*min*_. In this case, only two types of LRM models (i.e., LRM-1 and LRM-2) and two types of GAM models (GAM-1 and GAM-2) are compared to assess time series of local *P.vivax* infections. Given a set of candidate models for the data, the preferred model is the one with the minimum Akaike information criterion (AIC) and Bayesian information criterion (BIC) values. Table 2 shows the comparisons of LRM and GAM models with respect to local infections of *P.vivax* in Tengchong County. First, it can be observed that the nonlinear GAM models have better performances than corresponding LRM models. For example, GAM-1 (resp., GAM-2) model is better than LRM-1 (resp., LRM-2) model with smaller values of AIC, BIC and mean squared error (MSE), and larger *R* ^2^ and percentage of deviance explain. Second, even though some meteorological factors have strong correlations, the LRM-1 (resp., GAM-1) model, which involves more meteorological factors, has smaller AIC value but larger BIC value than the LRM-2 (resp., GAM-2) model. when fitting models, more parameters may result in overfitting. Both AIC and BIC rewards goodness of fit, but they also attempt to resolve the overfitting problem by introducing a penalty that is an increasing function of the number of estimated parameters (NUM). However, the penalty term in BIC is larger than in AIC. Therefore, we cannot determine which model between LRM-1 and LRM-2 (resp., between GAM-1 and GAM-2) is better.

**Table 1:** The Pearson coefficient matrix between time series of meteorological factors in Tengchong County

	*T*_*avg*_	*T*_*min*_	*T*_*max*_	*R*	*H*_*avg*_	*H*_*min*_
*T*_*avg*_	1					
*T*_*min*_	0.98	1				
*T*_*max*_	0.94	0.86	1			
*R*	0.662	0.73	0.47	1		
*H*_*avg*_	0.63	0.75	0.36	0.72	1	
*H*_*min*_	0.72	0.83	0.45	0.80	0.96	1

**Table 2:** The comparison of LRM and GAM models with respect to local *P. vivax* infections

Model	linear	df	NUM	AIC	BIC	MSE	R/squ	Deviance explain %
LRM-1	Y	9.00	8	208.22	231.30	0.43	0.34	33.80
GAM-1	N	18.31	8	197.84	244.80	0.31	0.45	45.30
LRM-2	Y	6.00	5	211.53	226.92	0.47	0.29	29.40
GAM-2	N	10.02	5	206.57	232.26	0.41	0.36	35.50

*df* residual degrees-of-freedom, *NUM* the number of parameters in the model, *AIC* Akaike information criterion, *BIC* Bayesian information criterion, *MSE* Mean squared error, *R/squ* the coefficient of determination *R* ^2^

### The GAM models with vectorial capacity

**Fig. 3:**
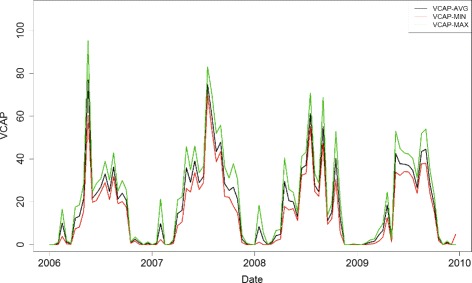
The vectorial capacity calculated using various temperatures *T*_*min*_, *T*_*avg*_, and *T*_*max*_ in Tengchong County. The notion of vectorial capacity is defined as the number of potentially infective contacts an individual person makes, through vector population, per unit time

Based on Eq. 1, the estimation of vectorial capacity *V* involves both temperature *T* and rainfall *R* in Tengchong. Accordingly, three VCAP values *V*_*min*_, *V*_*avg*_, and *V*_*max*_ can be calculated using different temperatures *T*_*min*_, *T*_*avg*_, and *T*_*max*_ (see Fig. 3). It can be observed that the VCAP curves have similar temporal patterns to the time series of *P. vivax* incidences in Tengchong. Since GAM models is superior to LRM models (see Table 2), to evaluate the effects of VCAP on local *P. vivax* infections, only GAM models are used to conduct the comparison (i.e., the GAM-V-MIN, GAM-V-AVG, and GAM-V-MAX models), where a linear trend (*a*+*bt*) is combined with VCAP to approximate the declining trend observed from time series decomposition in Tengchong County. Table 3 shows the performance of the three models with respect to local infections of *P. vivax* in Tengchong. Comparing with the LRM and GAM models in Table 2, all the three models have much better performance, representing that VCAP is more suitable to explain the risk of local infection. Moreover, the parametric coefficients show that all variables *V*_*min*_, *V*_*avg*_, *V*_*max*_, *H*_*avg*_, and *I*_*P*.*v*_ strongly support these models with significance level *p*<0.01.

**Table 3:** The performance of VCAP-associated GAM models with respect to local *P. vivax* infections

Model	linear	df	Num	AIC	BIC	MSE	R/squ	Deviance explain %
GAM-V-AVG	N	7.84	4	143.83	161.70	0.34	0.455	50.0
GAM-V-MIN	N	7.20	4	146.03	162.44	0.36	0.433	47.5
GAM-V-MAX	N	8.18	4	141.87	160.51	0.33	0.472	51.8

*df* residual degrees-of-freedom, *NUM* the number of parameters in the model, *AIC* Akaike information criterion, *BIC* Bayesian information criterion, *MSE* Mean squared error, *R/squ* the coefficient of determination *R* ^2^

### Model selection for estimating vectorial capacity

**Fig. 4:**
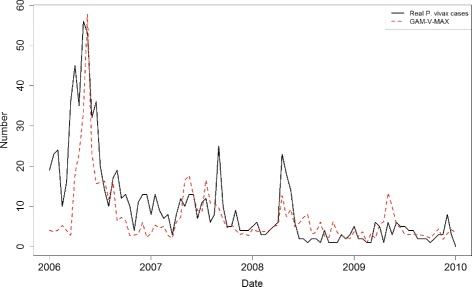
The fitting result of GAM-V-MAX model with respect to the real number of *P. vivax* infections

The results in Table 3 can also determine which temperature among *T*_*min*_, *T*_*avg*_, and *T*_*max*_ is better for estimating the transmission potential of *P. vivax* in Tengchong. This is critical for the prediction of malaria outbreaks. It can be observed that the GAM-V-MAX model performs slightly better than the other two models. To further evaluate the model, we conduct the deviance analysis. The null hypothesis is that the three models fit the data equally well, and the alternative hypothesis is that the GAM-V-MAX model is superior. Here the F-statistic is 4.34 and the associated *P*-value is 0.03. The result provides very clear evidence that the GAM-V-MAX model involving maximum temperature is superior to the GAM-V-MIN and GAM-V-AVG models. Figure 4 shows the estimated curve of *P. vivax* infections based on the GAM-V-MAX model. It can be observed that most periods with high risk of infection can be estimated by the GAM-V-MAX model.

**Fig. 5:**
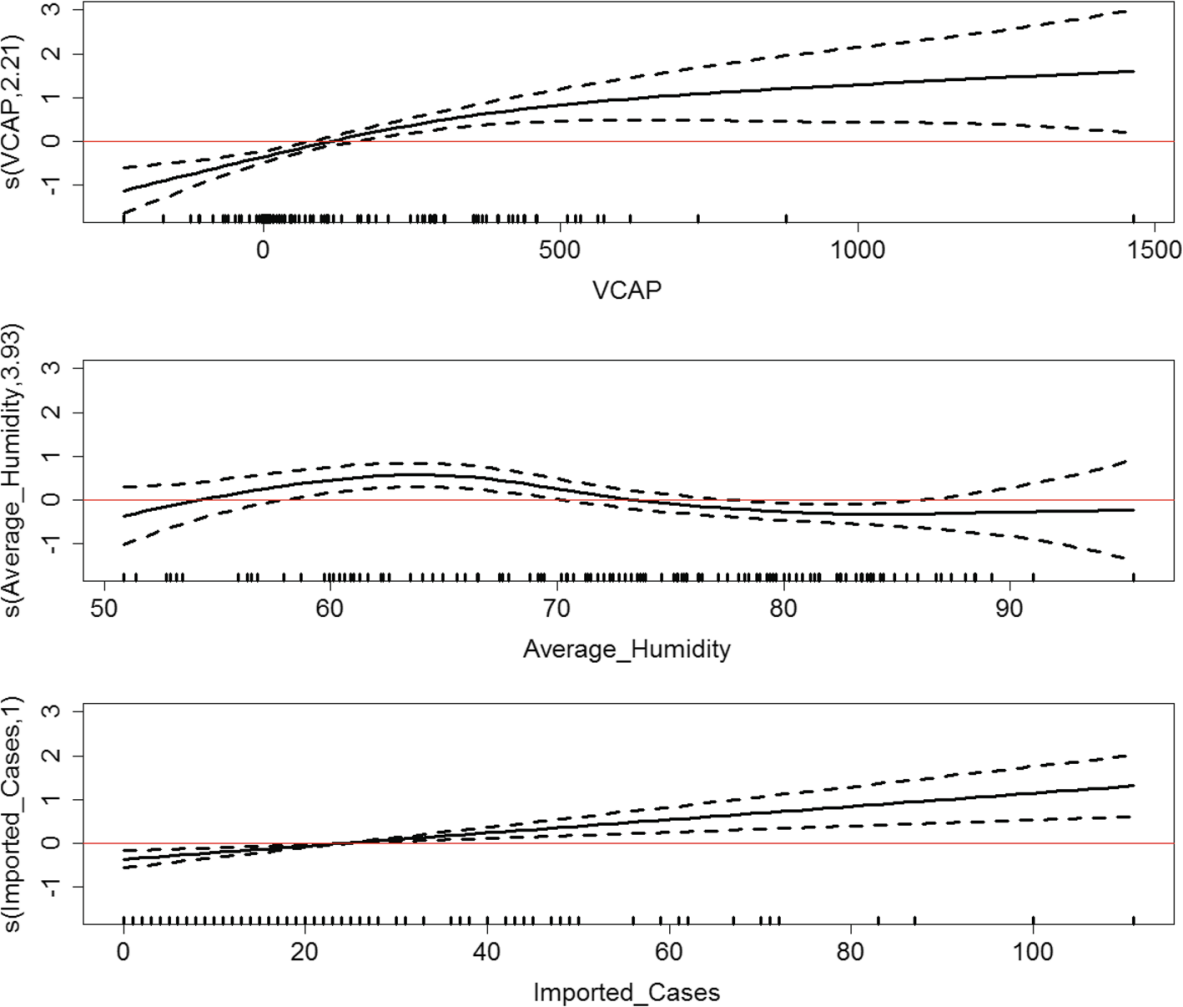
The estimated nonparametric smooths of VCAP, average humidity, and imported cases from the GAM-V-MAX model. The smooth of imported cases indicates that the true relationship between the number of local infections and that of imported cases is linear. The smooth of VCAP indicates that for lower VCAP the number of local infections increases at a constant rate but after passing a threshold value of VCAP the rate begins to decrease. While the relationship with average humidity is not significant in most regions

Figure 5 shows the estimated nonparametric smooths of VCAP, average humidity, and imported cases from the GAM-V-MAX model. Regions where the confidence bands (i.e., the dot lines) enclose the horizontal red line indicates corresponding values where the overall pattern is not significant. The smooth of imported cases indicate that the true relationship between the number of local infections and that of imported cases is linear. However, the estimated degrees of freedom (edf) for VCAP and average humidity is larger than 1, which indicate possible deviations from linearity. It can be observed from the Fig. 5 that the relationship with VCAP may be nearly linear: it appears that for lower VCAP the number of local infections increases at a constant rate but after passing a threshold value of VCAP the rate begins to decrease. On the other hand, the relationship with average humidity is not significant in most regions, except for the region between 60 and 70. In this region, the relationship with average humidity seems to be quadratic.

## Discussion

Human population movement (HPM) have been cited amongst the significant causes of the failure of the Global Malaria Eradication Programme fifty years ago [30, 31]. Human population movement from high to low or non malaria-endemic areas can result in imported infections, which may further trigger onward transmission [7–10]. Moreover, HPM patterns and the risk of malaria transmission vary substantially across spatial and temporal scales, socioeconomic sub-groups, and motivation for travel. Therefore, strategic control and elimination planning requires quantitative information on HPM patterns and the translation of these into parasite dispersion [32]. Extensive studies have been conducted attempting to quantify HPM patterns. For example, Wesolowski et al. quantified the impact of human mobility on malaria in Kenya using mobile phone data [10]; Menach et al. investigated the travel risk and malaria importation between Zanzibar and mainland Tanzania using mobile phone data and ferry traffic [33]; Tatem et al. proposed a method for targeting of interventions using surveillance data, satellite imagery and mobile phone call records to support elimination planning in Namibia [34]; Pindolia et al. have investigated the demographics of human movement and migration patterns in East Africa based on national population censuses and household surveys [35]. In high incidence areas of malaria, such census-style approaches have been verified to be helpful for malaria intervention and control. For China, which is about to reach the stage of malaria elimination and has only small number of imported cases, the above-mentioned approaches seem to be too resource-consuming and rough to characterize specific imported cases. In this paper, the seasonality analysis on time series of *P. vivax* incidences show that different patterns of human cross-border activity may result in different temporal patterns of *P. vivax* infections. Therefore, it would be helpful to perform active surveillance on motivations of human cross-border activity so as to plan targeted intervention strategies on imported cases.

Recently, together with the significant change of social and economic status, the corresponding malaria control strategies in Yunnan Province have also been changed. Besides traditional passive surveillance and vector controls, active surveillance and intervention have also been introduced, particularly in regions with high risk of infection. As compared with passive surveillance, active surveillance is much more ambitious with the aim of discovering every infection or imported case. Local CDC and surveillance agencies visit villages house by house to identify high risk populations. In this case, active surveillance is extremely time-consuming and requires massive experienced public health workers. However, human resources are very limited particularly in remote border area. For example, Tengchong has 18 towns, 205 villages and about 167 964 households distributed in a wide area of 5 845 square kilometers. However, in Tengchong CDC, less than 10 workers/investigators are available to perform active surveillance. The seasonality analysis for different counties can help frontier workers to perform active surveillance at the right place and time. For example, at Tengchong County, appropriate education and early warning can be conducted before peoples go to Myanmar once a year, while household survey can be implemented after they come back.

It is a particular challenge for malaria elimination at the China-Myanmar border area, especially when remote border areas of Myanmar have weak infrastructure and poor quality of treatments. In China, there is a sound surveillance system (CISDCP) for infectious diseases, where malaria incidences are reported from public health agencies everyday. However, in Myanmar, the surveillance and reporting system is too weak to collect sufficient information about the true malaria situation [36]. The weakness of health systems in Myanmar includes the limited capacity of local microscopists, incomplete coverage of surveillance for all communities and the lack of data reporting and management systems [19]. Because the meteorological factors are similar at the China-Myanmar border area, it is possible to use the same meteorological factors to assess the potential risk of malaria infection on both sides. In this paper, we have evaluated that the vectorial capacity (*V*_*max*_) calculated by maximum temperature (*T*_*max*_) and rainfall (*R*) is more suitable to fit the local *P. vivax* incidences in Tengchong County. Without further improvement of health systems in Myanmar, the GAM-V-MAX model could temporarily be used to estimate the risk of infection at the border area. On the other hand, detailed case studies on imported cases in China can also provide valuable malaria situations in Myanmar from which they came back.

## Conclusion

In this paper, we have assessed the risk of *P. vivax* transmission in Tengchong County, Yunnan Province, which is at the border area of China and Myanmar. First, different patterns of imported cases are decomposed for Tengchong and Ruili counties based on the seasonal and trend decomposition using Loess method. The seasonal patterns can provide useful information for active surveillance and further investigation of human cross-border movement, which is one of the biggest challenges for nationwide elimination of malaria in China. Second, the effect of meteorological factors on local risk of *P. vivax* infections is investigated using various linear regression models and generalized additive models. By comparison, it has been verified that the notion of vectorial capacity (VCAP) together with the number of imported cases is significant indicator to fit the number of local *P. vivax* incidences in Tengchong. Moreover, the maximum temperature has been assessed to be more suitable for estimating VCAP than the minimum and average temperature. Finally, the strong linear relationship with VCAP and imported cases indicates that to achieve effective malaria intervention, it is critical to perform active surveillance on the number of imported cases before the high risk season comes.

## Additional file

Additional file 1 Multilingual abstracts in the five official working languages of the United Nations. (PDF 381 kb)

## Abbreviations

CISDCP: China information system for disease control and prevention
GAM: Generalized additive model
HPM: Human population movement LRM: Linear regression model
MMP: Mobile and migrant population
NMEP: National malaria elimination programme
VCAP: Vectorial capacity
