# Supplementary material for: Risk assessment of malaria transmission at the border area of China and Myanmar
Source: Infect Dis Poverty. 2017 Jul 6;6:108. doi: 10.1186/s40249-017-0322-2 (PMC5499046; doi:10.1186/s40249-017-0322-2)
Supplement: Additional file 1 — Multilingual abstracts in the five official working languages of the United Nations. (PDF 381 kb) [file 40249_2017_322_MOESM1_ESM.pdf]

Translation of the abstract into the five official working languages of the United Nations

تقييم مخاطر انتقال الملاريا في المنطقة الحدودية بين الصين وميانمار

بن يون شي، جينشين تشنغ، هونغجون تشيو، غوو جينغ يانغ، شانغ شيا، شياو-نونغ تشو

الملخص

الخلفية: من أجل تحقيق هدف القضاء على الملاريا أطلقت الحكومة الصينية البرنامج الوطني للقضاء على الملاريا في 2010 غير انه نتيجة لتزايد التحركات السكانية عبر الحدود لا تزال مخاطر الإصابة بالملاريا الواردة من الخارج موجودة في المناطق الحدودية للصين مما يؤدي إلى تهديد محتمل بالانتقال المحلي للمرض وتركز هذه الورقة على تقييم حوادث الإصابة بالبلازمود النشيط (المتصورة النشيطة) التي جرت في تينغتشونغ بمقاطعة يونان في مناطق الحدودية بين الصين وميانمار.

الأساليب: تم تجميع بيانات التسلسل الزمني لحوادث الإصابة بالبلازمود النشيط في تينغتشونغ في الفترة من 2006 إلى 2010 من مركز المعلومات الصينية لمكافحه الامراض والوقاية منها على شبكة الإنترنت والتي تم فصلها في سلسله زمنيه من الحالات الواردة من خارج الصين والحالات المحلية وتم أولا عمل متابعة التحلل الموسمي والاتجاه في السلاسل الزمنية للحالات الواردة من الخارج باستخدام طريقه الرسوب وبعد ذلك تم تقييم تأثير العوامل المناخية علي الانتقال المحلي للبلازمود النشيط باستخدام نماذج الانحدار الخطي ونماذج الجمع المعمم وخصيصا تم استخدام مفهوم القدرة التصويرية لتقدير إمكانيات الانتقال في المواقع المختلفة استنادا إلي درجات الحرارة ومعدل هطول الامطار المحصلة من أداره الأرصاد الجوية الصينية.

النتائج: بالمقارنة مع مقاطعه رويلي فإن النمط الموسمي لحالات الإصابة الواردة من الخارج في تنغتشونغ يعتبر مختلفا حيث أنه في تينغتشونغ لا يوجد سوي ذروه واحده بينما يوجد ذروتين للمرض كل عام في رويلي ذروتين وقد يعزي ذلك إلى اختلاف سلوكيات الشعوب عبر الحدود في الموقعين وباستخدام القدرة التصويرية مع الحالات الواردة من الخارج ومتوسط الرطوبة فإنه من الممكن ان يتم تفسير حوادث الإصابة المحلية بالبلازمود النشيط بشكل جيد من خلال نماذج الانحدار الخطي ونماذج الجمع المعمم وعلاوة علي ذلك يتم التحقق من الحد الأقصى لدرجه الحرارة اليومية لتكون أكثر ملاءمة لحساب القدرة التصويرية لتقدير إمكانيات الانتقال من الحد الأدنى ومتوسط درجه الحرارة في مقاطعه تنغتشونغ.

الإستنتاج: لتحقيق القضاء على الملاريا في الصين فإن نتائج التقييم في هذه الورقة ستوفر مزيدا من التوجيه في مجال المراقبة النشطة للملاريا ومكافحتها في المناطق الحدودية بين الصين وميانمار.

Translated from English version into Arabic by Mohamed R. Habib

中国和缅甸边境地区疟疾传播的风险评估

史本云，郑金鑫，邱洪君，杨国静，夏尚，周晓农

摘要

引言：为了实现疟疾消除的目标，中国政府于 2010 年启动了中国疟疾消除计划。然而，由于越来越多的跨境人口流动，中国边境地区仍然存在输入性疟疾的风险，从而对疟疾的本地传播构成潜在威胁。本文关注的重点是评估中缅边境云南腾冲地区间日疟的传播风险。

方法：我们从中国疾病预防控制信息系统采集了从 2006 年至 2010 年腾冲地区间日疟的日发病数据，包括输入病例和本地病例时间序列。 首先，我们使用 Loess 法对输入病例的时间序列进行季节性和趋势分解。 然后，使用线性回归模型（LRM）和广义加法模型（GAM）评估气候因素对间日疟本地传播的影响。具体地，我们使用了媒介能量（VCAP）的概念，利用从中国气象局收集的温度和降雨量等数据，评估不同地区间日疟的传播潜力。

结果：腾冲县和瑞丽县的输入病例季节性有很大的不同：腾冲县的输入病例每年只有一个高峰，而瑞丽县每年有两个峰值。这可能是由两地人口的不同的跨境行为导致的。通过线性回归模型和广义加法模型，我们发现媒介能量、输入病例以及平均湿度能够更好地解释间日疟的本地发病率。而且，腾冲县的日最高温度比日最低温度和平均温度更适合于估算媒介能量。
结论：本文的评估方法和结果将为中国和缅甸边境地区疟疾疫情的主动监测和控制提供科学的依据和思路。

Translated from English version into Chinese by Benyun Shi

Évaluation de risque de transmission du paludisme à la frontière de la Chine et du Myanmar

Benyun Shi, Jinxin Zheng , Hongjun Qiu, Guo-Jing Yang, Shang Xia, and Xiao-Nong Zhou

Résumé

Contexte: Afin d'atteindre l'objectif de l'élimination du paludisme, le gouvernement Chinois a lancé le Programme National de Lutte contre le Paludisme en 2010. Toutefois, en raison de l'augmentation des mouvements transfrontaliers de population, le risque d'importation de cas de paludisme existe toujours dans les zones frontalières de la Chine, d'où un risque de transmission locale. Le présent article a pour objet d'évaluer les cas à *Plasmodium vivax* à Tengchong dans la Province de Yunnan à la frontière de la Chine et du Myanmar.
Méthodes: Les séries chronologiques d’infection à *P. vivax* dans le Comté de Tengchong de 2006 à 2010 ont été fournies par le Système d'Information de Chine pour le Contrôle et la Prévention des Maladies, qui sont ensuite subdivisées en série chronologique de cas importés et locaux. D'abord, la décomposition est effectuée suivant les variations saisonnières et les séries de cas importés en utilisant la méthode de Loess. Puis, l'impact des facteurs climatiques sur la transmission locale à *P. vivax* est évalué à l'aide de modèles de régression linéaire (MRL) et de modèles additifs généralisés (MAG). Concrètement, la notion de capacité vectorielle (CAPV) est utilisée pour estimer le risque de transmission à *P. vivax* dans des endroits différents. La CAPV est calculée en fonction de la température et des précipitations recueillies auprès de l'Administration Météorologique Chinoise.
Résultats: En comparaison avec le Comté de Ruili, la tendance saisonnière de cas importés à Tengchong est différente: Tengchong ne présente qu'un seul pic, alors que Ruili a deux pics chaque année. Ceci pourrait être dû aux différents comportements transfrontaliers des peuples en deux endroits. La capacité vectorielle, ensemble avec les cas importés et l'humidité moyenne peuvent bien expliquer les cas locaux d'infection à *P. vivax* à partir des méthodes de MAG et CAPV. En outre, la température maximale quotidienne est vérifiée à être plus appropriée pour calculer la CAPV que la température minimale ou moyenne dans le Comté de Tengchong.
Conclusion: Afin de parvenir à l'élimination du paludisme en Chine, les résultats de cette évaluation fourniront de nouvelles orientations dans la surveillance active et le contrôle du paludisme dans les zones frontalières de la Chine et du Myanmar.

Translated from English version into French by Kokouvi Kassegne

Оценка риска распространения малярии в пограничных районах Китая и Мьянмы

Benyun Shi, Jinxin Zheng , Hongjun Qiu, Guo-Jing Yang, Shang Xia, and Xiao-Nong Zhou

Аннотация

Введение: В 2010 году правительство КНР запустило Национальную Программу по ликвидации малярии. Однако, в результате увеличения случаев пересечения границы между близлежащими странами и КНР, риск возникновения случаев заражения малярией в результате потенциальной угрозы местного распространения до сих пор сохраняется. Данное исследование главным образом фокусируется на оценке зарегистрированных случаев появления трехдневной малярии или *Plasmodium vivax* в городе Тенгчонг, провинции Юньнань, граничащем с Мьянмой.

Методы исследования: Динамика заболеваемости населения г.Тенгчонг *P. vivax* в период с 2006 по 2010 гг. была взята с размещенной в интернете Информационной Системы Китая по контролю и предупреждению заболеваний, которая впоследствии была разделена по конкретным динамикам привнесенных и местных случаев заболевания. Прежде всего, там представлена сезонная динамика деструкции привнесенных случаев заболевания, которая была составлена, используя алгоритм LOESS или Метод локальных полиномиальных регрессий. Затем, воздействие климатических факторов на местную передачу *P. vivax* оценивается с помощью линейных регрессионных моделей (LRM) и обобщенных аддитивных моделей (GAM). В частности, понятие VCAP используется для оценки возможности передачи *P. vivax* в разных местах, которое рассчитывается на основании показателей температуры и осадков, собранных Китайской метеорологической администрацией.

Результаты исследования: По сравнению с городским уездом Жуйли в Китае, характер сезонных изменений привнесенных случаев заражения в г.Тенгчонг значительно отличается: в Тенгчонг был лишь один скачок, в то время как в Жуйли было два скачка в год. Что может быть обусловлено различиями в поведении людей, живущих в приграничных районах обоих населенных пунктов. Все вместе: способность быть переносчиком инфекции (VCAP), привнесенные случаи и средний уровень влажности – могут послужить хорошим объяснением местных инцидентов заражения *P. vivax*, с помощью метода локальных полиномиальных регрессий и линейной регрессионной модели. Кроме того, максимальная дневная температура воздуха, как было проверенно ранее, является более подходящей для расчета VCAP, чем минимальная и средняя температуры в Тенгчонг и Жуйли.

Выводы: Результаты оценивания в данном исследовании послужат дополнительным ориентиром для активного наблюдения и контроля малярии в пограничных районах Китая и Мьянмы, с целью ликвидации данного заболевания в КНР.

Translated from English version into Russian by Hao-Qi Zhang

Evaluación del riesgo de transmisión de la malaria en la zona fronteriza de China y Myanmar

Benyun Shi, Jinxin Zheng , Hongjun Qiu, Guo-Jing Yang, Shang Xia, and Xiao-Nong Zhou

Abstracto

Trasfondo: Con el fin de lograr el objetivo de la eliminación de la malaria, el gobierno chino lanzó el Programa Nacional de Eliminación de la Malaria en 2010. Sin embargo, como resultado del aumento de los movimientos transfronterizos de población, el riesgo de casos importados de malaria sigue existiendo en las zonas fronterizas de China , resultando en una amenaza potencial de transmisión local. El objetivo de este trabajo es evaluar las incidencias de *Plasmodium vivax* en Tengchong, provincia de Yunnan, en las zonas fronterizas de China y Myanmar.

Métodos: Las series cronológicas de incidencias de *P. vivax* en Tengchong de 2006 a 2010 son recopiladas del Sistema de Información de China para el Control y la Prevención de Enfermedades, los que luego se separan en series temporales de casos importados y locales. En primer lugar, la descomposición estacional y de tendencias se realiza en series temporales de casos importados utilizando el método de Loess. Luego, se evalúa el impacto de los factores climáticos en la transmisión local de *P. vivax* utilizando modelos de regresión lineal (MRL) y modelos aditivos generalizados (MAG). Específicamente, la noción de capacidad vectorial (CAPV) se utiliza para estimar el potencial de transmisión de *P. vivax* en diferentes lugares, la cual se calcula sobre la basado en temperatura y las precipitaciones recolectadas de la Administración Meteorológica de China.

Resultados: Comparando con el condado de Ruili, el patrón estacional de los casos importados en Tengchong es diferente: Tengchong tiene solamente un pico, mientras que Ruili tiene dos picos durante cada año. Esto puede deberse a los diferentes comportamientos transfronterizos de los pueblos en dos lugares. La capacidad vectorial, junto con los casos importados y la humedad media, pueden explicar bien las incidencias locales de *P. vivax* a través de los métodos MRL y MAG. Además, se verifica que la temperatura máxima diaria es más adecuada para calcular CAPV que la temperatura mínima y media en el condado de Tengchong.

Conclusión: Para lograr la eliminación de la malaria en China, los resultados de la evaluación en este documento proporcionarán más orientación en la vigilancia activa y el control de la malaria en las zonas fronterizas de China y Myanmar.

Translated from English version into Spanish by Laura C Vicente Rodriguez
